# A genome-wide cross-trait analysis identifies genomic correlation, pleiotropic loci, and causal relationship between sex hormone-binding globulin and rheumatoid arthritis

**DOI:** 10.1186/s40246-023-00528-x

**Published:** 2023-08-29

**Authors:** Yuan Jiang, Qianwen Liu, Lars Alfredsson, Lars Klareskog, Ingrid Kockum, Xia Jiang

**Affiliations:** 1 Department of Clinical Neuroscience, Center for Molecular Medicine, Karolinska Institutet, Visionsgatan 18, 171 77 Solna, Stockholm, Sweden; 2 Institute of Environmental Medicine, Karolinska Institutet, Solna, Stockholm, Sweden; 3 Department of Medicine, Karolinska Institutet, Solna, Stockholm, Sweden; 4 Department of Epidemiology and Biostatistics, Institute of Systems Epidemiology, and West China-PUMC C. C. Chen Institute of Health, West China School of Public Health and West China Fourth Hospital, Sichuan University, Chengdu, Sichuan China

**Keywords:** Sex hormone-binding globulin, Rheumatoid arthritis, Genome-wide cross-trait analysis, Mendelian randomization analysis

## Abstract

**Background:**

Our study aims to investigate an intrinsic link underlying sex hormone-binding globulin (SHBG) and rheumatoid arthritis (RA), which remains inconclusive in observational settings.

**Methods:**

Summary statistics were collected from the largest GWAS(s) on SHBG adjusted for BMI (SHBG_adj_BMI; *N*_overall_ = 368,929; *N*_men_ = 180,094; *N*_women_ = 188,908), crude SHBG (*N*_overall_ = 370,125; *N*_men_ = 180,726; *N*_women_ = 189,473), and RA (*N*_case_ = 22,350; *N*_control_ = 74,823). A genome-wide cross-trait design was performed to quantify global and local genetic correlation, identify pleiotropic loci, and infer a causal relationship.

**Results:**

Among the overall population, a significant global genetic correlation was observed for SHBG_adj_BMI and RA ($r_{{\text{g}}}$ = 0.11, *P* = 1.0 × 10^−4^) which was further supported by local signal (1q25.2). A total of 18 independent pleiotropic SNPs were identified, of which three were highly likely causal variants and four were found to have effects on both traits through gene expression mediation. A putative causal association of SHBG_adj_BMI on RA was demonstrated (OR = 1.20, 95% CI = 1.01–1.43) without evidence of reverse causality (OR = 0.999, 95% CI = 0.997–1.000). Sex-specific analyses revealed distinct shared genetic regions (men: 1q32.1-q32.2 and 5p13.1; women: 1q25.2 and 22q11.21-q11.22) and diverse pleiotropic SNPs (16 in men and 18 in women, nearly half were sex-specific) underlying SHBG_adj_BMI and RA, demonstrating biological disparities between sexes. Replacing SHBG_adj_BMI with crude SHBG, a largely similar yet less significant pattern of results was observed.

**Conclusion:**

Our cross-trait analysis suggests an intrinsic, as well as a sex-specific, link underlying SHBG and RA, providing novel insights into disease etiology.

**Supplementary Information:**

The online version contains supplementary material available at 10.1186/s40246-023-00528-x.

## Background

Human autoimmune disorders often present sex-specific characteristics. In rheumatoid arthritis (RA), women account for more than 70% of all cases [1] whom also exhibit higher disease activity, worse functionality, and more severe comorbidities [2]. Epidemiological studies have linked hormone alterations with RA onset. For instance, post-menopause and postpartum, characterized by declined estrogen levels, have been linked to an increased risk of developing RA. Conversely, periods during pregnancy and breastfeeding, featured by increased estrogen levels, have been associated with a reduced risk of RA [3]. However, the potential pathogenic role of hormones in RA remains largely uncharacterized.

Sex hormone-binding globulin (SHBG) acts as a carrier of sex steroids and regulates the extent to which these hormones are delivered to body tissues [4], mediating the relationship underlying various endocrine organs and pathophysiology of diseases [5]. Clarifying the role of SHBG in RA provides novel insights into not only disease etiology but perhaps also sex disparity. So far, studies investigating the SHBG-RA relationship remain sparse with largely inconsistent findings [6–8]. For example, a small-sized case–control study (55 RA and 50 controls) reported SHBG levels to be significantly lower in female cases than in controls, both pre-menopausal (34.8 ± 10.0 vs. 58.4 ± 17.9 nmol/L) and post-menopausal (35.5 ± 10.7 vs. 44.9 ± 7.1 nmol/L) [7], while another case–control study (120 RA and 518 controls) did not support such a difference (post-menopausal: 57.6 ± 26.9 vs. 57.3 ± 27.3 nmol/L) [6]. As for men, SHBG did not seem to differ significantly according to results from a case–control study involving 104 male cases and 99 age- and sex-matched controls [8]. Except for insufficient power, discrepancies in these results could also derive from bias, confounding, or reverse causality which are common in conventional epidemiological studies. Mendelian randomization (MR) analysis narrows the gap by elucidating a putative exposure-outcome causal association, using genetic variants (single nucleotide polymorphisms, SNPs) as instrumental variables [9]. Applying a two-sample MR framework, a positive causal association of circulation SHBG with RA has been reported (OR = 1.003; 95% CI = 1.000–1.007) [10]. Nevertheless, this MR used only a handful of instruments (*N*_IV_ = 13) and a small number of RA cases (*N*_RA_ = 4017), substantially restricting the robustness of the results.

**Fig. 1:**
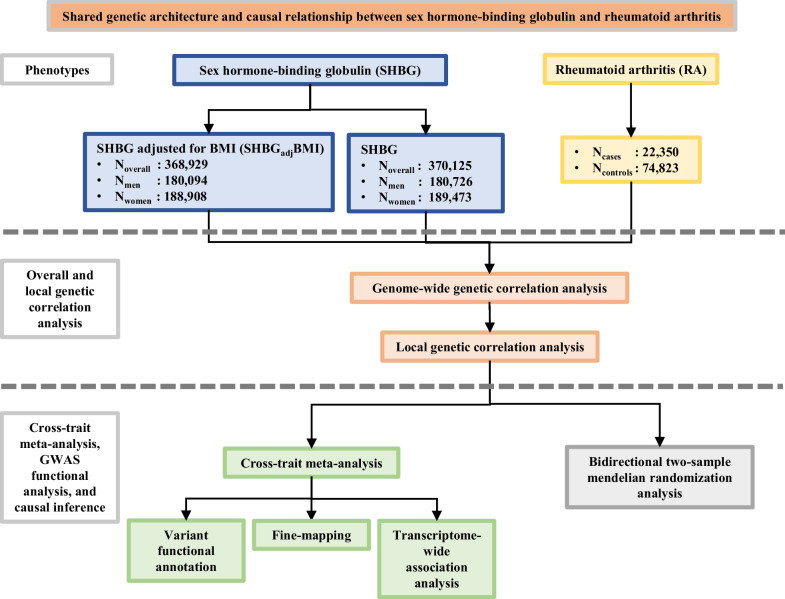
Flowchart of the overall study design. We investigated the shared genetic architecture underlying sex hormone-binding globulin (SHBG) and rheumatoid arthritis (RA). We first quantified the genome-wide genetic correlation between SHBG and RA; we then estimated local genetic correlations by partitioning the genome into linkage-disequilibrium independent blocks. Next, we identified potential pleiotropic loci contributing to both traits and conducted functional annotation for these loci. We also performed fine-mapping and transcriptome-wide association analysis to provide biological insight. Finally, to make a causal inference, we conducted a bidirectional Mendelian randomization

The increasing availability of genetic data produced by large-scale genome-wide association studies (GWAS) enables the utilization of a compiled analytical strategy named genome-wide cross-trait analysis. This analysis features several analytical aspects and permits the quantification of shared and distinct etiology underlying complex traits. Our study, therefore, aims to extend previous findings by implementing a comprehensive genome-wide cross-trait approach, leveraging summary statistics of the hitherto largest GWAS(s) conducted for SHBG and RA. In addition to genetic data of overall circulating SHBG (*N*_overall_ = 370,125), data on sex-specific SHBG (*N*_men_ = 180,726, *N*_women_ = 189,473) and BMI-adjusted SHBG (SHBG_adj_BMI, *N*_overall_ = 368,929, *N*_men_ = 180,094, and *N*_women_ = 188,908) were further incorporated to detect sex disparity and to control for the confounding effect of BMI. We first quantified the global and local genetic correlation underlying SHBG and RA, and then identified potential pleiotropic loci affecting both traits. We conducted functional annotation of these loci and performed fine-mapping analysis and transcriptome-wide association study (TWAS) to provide biological insight. We finally evaluated a putative causal relationship. A flowchart of the overall study design is shown in Fig. 1.

## Methods

*SHBG GWAS* The latest and largest GWAS of circulating SHBG was conducted using UK Biobank (UKBB), which involved 370,125 participants of European ancestry. Genetic variants were imputed using the Haplotype Reference Consortium and the 1000 Genomes Project reference panel. In addition to the sample quality control metrics performed centrally by UKBB, white European ancestry was determined by *K*-means clustering combined with self-report. Genetic variants with minor allele frequency (MAF) > 1% as well as those that passed quality control in batches were involved. Association test was performed using a linear mixed model, adjusted for genotyping chip, age at baseline, and ten genetically derived principal components (PCs). Genome-wide significant index variants (*P* < 5 × 10^–8^) were identified using 1 Mb distance-based clumping with linkage disequilibrium (LD) < 0.05 across all variants [11].

To account for the confounding effect of body mass index (BMI), additional analysis was performed among 368,929 UKBB individuals, which consisted of individuals enrolled in the SHBG GWAS with BMI records. BMI was adjusted (SHBG_adj_BMI) along with the aforementioned variables. Sex-specific analyses were also performed (SHBG: *N*_men_ = 180,726, *N*_women_ = 189,473; SHBG_adj_BMI: *N*_men_ = 180,094, *N*_women_ = 188,908) [11].

Significant index SNPs discovered by these GWAS(s) were used as instrumental variables (IVs). For crude SHBG, 335 SNPs were identified, with corresponding figures of 188 for men and 176 for women. For SHBG_adj_BMI, 477 SNPs were identified, with corresponding figures of 251 for men and 271 for women. The characteristics of SHBG-associated (crude, BMI-adjusted, and sex-specific) index SNPs used for MR analysis are presented in (Additional file 1: Table S1–S6). The full sets GWAS summary statistics were also obtained and used for other genetic analyses.

*RA GWAS* The hitherto largest GWAS of RA was conducted by meta-analyzing 25 cohorts involving 22,350 cases and 74,823 controls of European ancestry. Genotype quality control, imputation, and case–control association analyses were conducted separately for each cohort. Quality control excluded samples with low call rate, closely related individuals, or outliers based on ancestries identified through PC analysis. Additionally, variants with low call rate, low MAF, or low Hardy–Weinberg *P* value were excluded. Genotyping imputation was performed using 1000 Genomes Phase 3 panel with minimum imputation accuracy (*r*^2^*)* of 0.3. SNPs associated with RA were detected assuming additive effects using logistic regression models, correcting for sex and PCs. Finally, effect sizes of the identical SNPs across all participating studies were combined using a fixed-effect inverse variance weighted meta-analysis [12]. Index SNPs were obtained with genome-wide significance (*P* < 5 × 10^–8^) using 1 Mb distance-based clumping with linkage disequilibrium (LD) < 0.05 across all variants after removing the Major Histocompatibility Complex (MHC) region. A total of 182 RA-associated index SNPs were identified, of which 175 were available and used as IVs in our bidirectional MR analysis. The full set of GWAS summary statistics was downloaded and used for other analyses. To the best of our knowledge, none of these 25 studies overlapped with participants in UKBB, our exposure GWAS(s) [12]. For all analyses, the human reference genome build 37 (hg19) was used.

### Statistical analysis

#### Genome-wide genetic correlation analysis

Genome-wide genetic correlation ($r_{{\text{g}}}$) was estimated using cross-trait LD-score regression (LDSC) [13, 14]. This algorithm quantifies the average sharing of genetic effects between pairs of traits that is independent of environmental confounders. On average, SNPs in high LD exhibit higher average χ2 statistics than SNPs in low LD in polygenic traits. Similarly, when analyzing traits with genetic correlation, the product of *z*-scores from two studies presents a comparable relationship with χ2 statistics for a single study. The algorithm is described in the formula as follows:

$$E_{{\left[ {\beta_{j}\gamma_{j}} \right]}} = \frac{{\sqrt {N_{1} N_{2} } r_{{\text{g}}} }}{M}l_{j} + \frac{{N_{{\text{s}}} r}}{{\sqrt {N_{1} N_{2} } }}$$

Among them, ß$_{j}$ and γ$_{j}$ represent the z-scores of SNP j on traits 1 and trait 2, $r_{{\text{g}}}$ represents the genetic covariance, $M$ represents the number of SNPs, $N_{1}$ and $N_{2}$ represent the sample sizes of each trait, $N_{{\text{s}}}$ represents the number of overlapping samples, ${\text{r}}$ represents the phenotypic correlation in overlapping samples, and $l_{j}$ represents the LD score. The estimates of genetic correlation range from − 1 to 1, with − 1 representing a completely negative correlation and 1 representing a completely positive correlation. The MHC region (chr6:28,477,797–33,448,354), known for its strong effects on autoimmune conditions and complex LD pattern, was excluded from this analysis. Given that analyses were repeated three times (overall and sex-specific populations), a Bonferroni-corrected *P* value of 0.05/3 was used to define statistical significance.

#### Local genetic correlation analysis

Genomic correlation collapses the effect of all SNPs across the whole genome. Even with a negligible global genetic correlation, there might be specific regions in the genome affecting both traits. We next quantified local genetic correlation using heritability estimation from summary statistics (ρ-HESS). The genome was partitioned into 1,703 LD-independent regions with an average size of 1.6 Mb. Firstly, genetic covariance was used to evaluate trait similarity within each LD-independent region under a fixed-effect model. Genetic covariance quantifies the covariation of traits on their original scales. To enable comparisons across different traits and genomic regions, genetic correlation was further employed to standardize covariation using the jackknife approach [15]. The MHC region was excluded from this analysis. A Bonferroni-corrected *P* value of 0.05/(1703 × 3) was used to define statistical significance, considering analyses across 1703 LD-independent regions for the overall and sex-specific populations.

#### Cross-trait meta-analysis

Genetic correlation suggests shared genetic components—either due to genetic variants having an independent effect on both traits (horizontal pleiotropy or pleiotropy) or genetic variants influencing one trait via its effect on the other (vertical pleiotropy or causality). To detect potential pleiotropic loci, we applied a cross-trait meta-analysis using cross-phenotype association analysis (CPASSOC) [16]. This algorithm integrates association evidence from multiple traits to detect variants affecting at least one trait. CPASSOC has several attractive features. In addition to allowing for sample overlap and relying only on summary statistics, it is compatible with trait heterogeneity effects, where a specific genetic variant may have varying magnitudes of effects (including different directions) on different trait [17]. Given the fact that trait homogeneity assumption is less likely to hold with multiple traits involved, test statistic *S*_Het_ (rather than *S*_Hom_) which improves statistical power in the presence of trait heterogeneous effects was therefore used to combine association evidence. We obtained independent top-associated loci applying PLINK LD-based clumping function (parameters: –clump-p1 5e−8 –clump-p2 1e−5 –clump-r2 0.2 –clump-kb 500). Significant pleiotropic SNPs were defined as variants with *P*_single-trait_ < 1 × 10^–5^ in both traits and *P*_CPASSOC_ < 5 × 10^–8^ in paired traits. These SNPs were further divided into four categories. First, a “known” pleiotropic SNP was one that reached genome-wide significance in both single traits (*P*_SHBG_ < 5 × 10^–8^, *P*_RA_ < 5 × 10^–8^, and *P*_CPASSOC_ < 5 × 10^–8^). These SNPs were naturally pleiotropic even without performing CPASSOC. Second, a “single-trait-driven” pleiotropic SNP was one that reached genome-wide significance in one of two single traits (*P*_SHBG_ < 5 × 10^–8^ or *P*_RA_ < 5 × 10^–8^ and *P*_CPASSOC_ < 5 × 10^–8^). Third, an “LD-tagged” pleiotropic SNP was one that, despite not reaching genome-wide significance in any single trait (5 × 10^–8^ < *P*_SHBG / RA_ < 1 × 10^–5^ and *P*_CPASSOC_ < 5 × 10^–8^), was in LD (*r*^2^ threshold = 0.2) with index SNPs (or any SNP located within ± 250 kb around the index SNPs) identified by single-trait GWAS(s). Finally, a “novel” pleiotropic SNP was of great interest to us, which was defined as those that neither reached genome-wide significance in any single trait nor in LD with previously identified SHBG- or RA-associated SNPs. Ensembl Variant Effect Predictor (VEP) was used to map pleiotropic SNPs to the nearest genes based on location [18].

#### Fine-mapping analysis

Index SNPs are not necessarily causal variants. We further identified a 99% credible set of causal variants through FM-summary method, a simplified Bayesian fine-mapping method using summary statistics. Briefly, each pleiotropic SNP and the variants within 500 kb around, extracted from the pooled results of cross-trait meta-analysis, were used as input for FM-summary. FM-summary then set a flat prior and generated a posterior inclusion probability (PIP) of a true trait/disease association for each variant using the steepest descent approximation [19, 20]. A 99% credible set is equivalent to sorting SNPs from the largest to the smallest PIPs and taking the cumulative sum of PIPs until it reaches at least 99%.

#### Transcriptome-wide association study

Cross-trait meta-analysis identifies pleiotropic loci affecting both traits without considering gene expression, while many pleiotropic loci influence complex traits by modulating gene expression levels. To identify relevant genes whose expression patterns vary across tissues, we performed a TWAS analysis. Imputable genes were provided by pre-trained joint-tissue imputation prediction models (GTEx v8) [21]. Gene-phenotype association analysis was performed by S-PrediXcan [21, 22]. We first performed a single-trait TWAS and then intersected these results based on gene-tissue pair to examine if they were shared across traits. Bonferroni correction was used considering the number of gene-tissue pairs tested in each trait and the analyses repeated in overall and sex-specific populations.

#### Bidirectional Mendelian randomization analysis

A two-sample bidirectional MR analysis was performed to identify a putative causal relationship. Inverse variance weighted (IVW) approach was used as our primary approach, assuming all IVs were valid, or the overall pleiotropy was balanced to zero [23]. A series of sensitivity analyses were conducted to validate model assumptions and to guarantee the robustness of our findings. The MR-Egger intercept test was used to reflect directional pleiotropy [24]. Weighted median approach was performed under the assumption that up to 50% of IVs contributing to analysis were invalid [25]. MR pleiotropy residual sum and outlier test (MR-PRESSO) was applied to detect outliers and obtain outlier-corrected effects [26]. Additionally, MR-PRESSO distortion test was performed to examine the discrepancies between the causal estimates before and after outlier correction [26]. IVW approach was further repeated by excluding palindromic SNPs (A/T or G/C SNPs introducing ambiguity into the identification of effect alleles) or pleiotropic SNPs (SNPs affecting phenotypes served as potential confounders of the SHBG-RA relationship, identified through LDLink, and violating the exclusion restriction assumption). Details of the excluded pleiotropic SNPs and their associated confounders are shown in Additional file 1: Table S1–S6. Direction of causality was inferred using Steiger test. Robust adjusted profile score (MR-RAPS) was further applied to validate the conformity of results. To test whether the causal estimates were driven by individual SNP, a leave-one-out analysis was performed with each SNP iteratively removed, and IVW applied using the remaining SNPs.

Statistical power was calculated using the non-centrality parameter of the test statistic as suggested by Brion et al. [27]. The proportion of variance explained by IVs was computed using the formula provided by Shim et al. [28]. Given analyses were repeated three times (overall and sex-specific populations), an assumed α of 0.05/3 was employed. The strength of the IVs was evaluated using the *F*-statistic.

All analyses were conducted with packages “TwoSampleMR”, “MRInstruments”, “MendelianRandomization”, “MR-PRESSO” and “mr.raps” in R v3.6.3. Given analyses were repeated three times (overall and sex-specific populations), a Bonferroni-corrected *P* value threshold of 0.05/3 was used to define statistical significance in MR. Additionally, a *P* value of < 0.05 was employed for suggestive significance.

### Ethics/consent statement

This was a secondary analysis of existing, publicly available summary-level GWAS data. The statement of ethics for each GWAS can be found elsewhere, approved by relevant ethics committees [11, 12].

## Results

### Genetic correlations between SHBG and RA

After correcting for multiple testing (*P* < 0.05/3), as shown in Table 1, we found a minimal shared genetic basis between crude SHBG and RA ($r_{{\text{g}}}$ = 0.05, *P* = 3.9 × 10^–2^). The effects remained null in both men (${\text{r}}_{{\text{g}}}$ = 0.05, *P* = 3.2 × 10^–2^) and women ($r_{{\text{g}}}$ = 0.04, *P* = 0.10), possibly due to the confounding effect of BMI as studies have found a decreased level of SHBG [29] but an increased risk of RA [30] among individuals who were overweight or obese. As expected, after adjusting for BMI, a positive genome-wide genetic correlation was observed for SHBG_adj_BMI and RA ($r_{{\text{g}}}$ = 0.11, *P* = 1.0 × 10^–4^), and this effect remained consistent in sex-specific analysis (men: $r_{{\text{g}}}$ = 0.07, *P* = 1.1 × 10^–3^, women: $r_{{\text{g}}}$ = 0.09, *P* = 2.0 × 10^–4^). Therefore, in our subsequent analysis, SHBG_adj_BMI was used as primary exposure, complemented by crude SHBG.

**Table 1:** Genome-wide genetic correlation between sex hormone-binding globulin and rheumatoid arthritis, excluding the MHC region

Trait	${\text{r}}_{{\text{g}}}$	SE	Z	*P*
*Overall*				
SHBG_adj_BMI	0.11	0.03	3.8	1.0 × 10^–4^
SHBG	0.05	0.02	2.06	3.9 × 10^–2^
*Men*				
SHBG_adj_BMI	0.07	0.02	3.26	1.1 × 10^–3^
SHBG	0.05	0.02	2.14	3.2 × 10^–2^
*Women*				
SHBG_adj_BMI	0.09	0.02	3.71	2.0 × 10^–4^
SHBG	0.04	0.03	1.65	0.10

$r_{{\text{g}}}$, Genetic correlation; SE, Standard error; SHBG_adj_BMI, Sex hormone-binding globulin adjusted for BMI; SHBG, Sex hormone-binding globulin

**Fig. 2:**
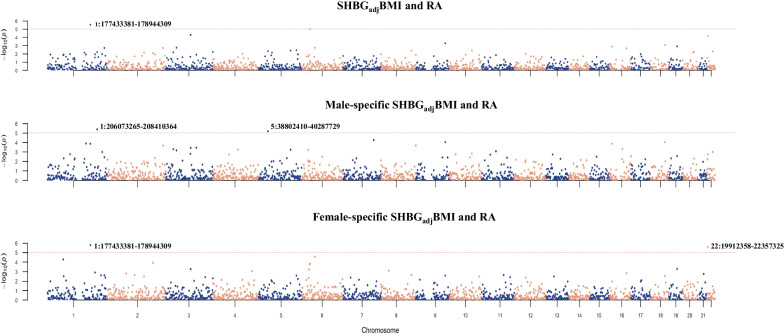
Local genetic correlation: BMI-adjusted sex hormone-binding globulin and rheumatoid arthritis (excluding MHC). *X*-axis represents chromosomes, *Y*-axis represents negative logarithm of *P* values, and each dot in the Manhattan plot represents a linkage-disequilibrium independent genomic region. Chromosomes and genomic regions for significant local genetic correlations are marked. MHC regions were excluded due to their complex LD pattern. SHBG_adj_BMI: sex hormone-binding globulin adjusted for BMI; RA: rheumatoid arthritis

Partitioning the whole genome into 1703 regions, we identified significant local genetic correlations for SHBG_adj_BMI with RA at one region (1q25.2) (Fig. 2). In addition, distinct genomic regions were identified in men (1q32.1-q32.2 and 5p13.1) and women (1q25.2 and 22q11.21-q11.22).

Analysis of crude SHBG with RA presented insignificant results, further demonstrating the effectiveness of focusing on SHBG_adj_BMI (Additional file 2: Fig. S1).

### Cross-trait meta-analysis of SHBG and RA

**Fig. 3:**
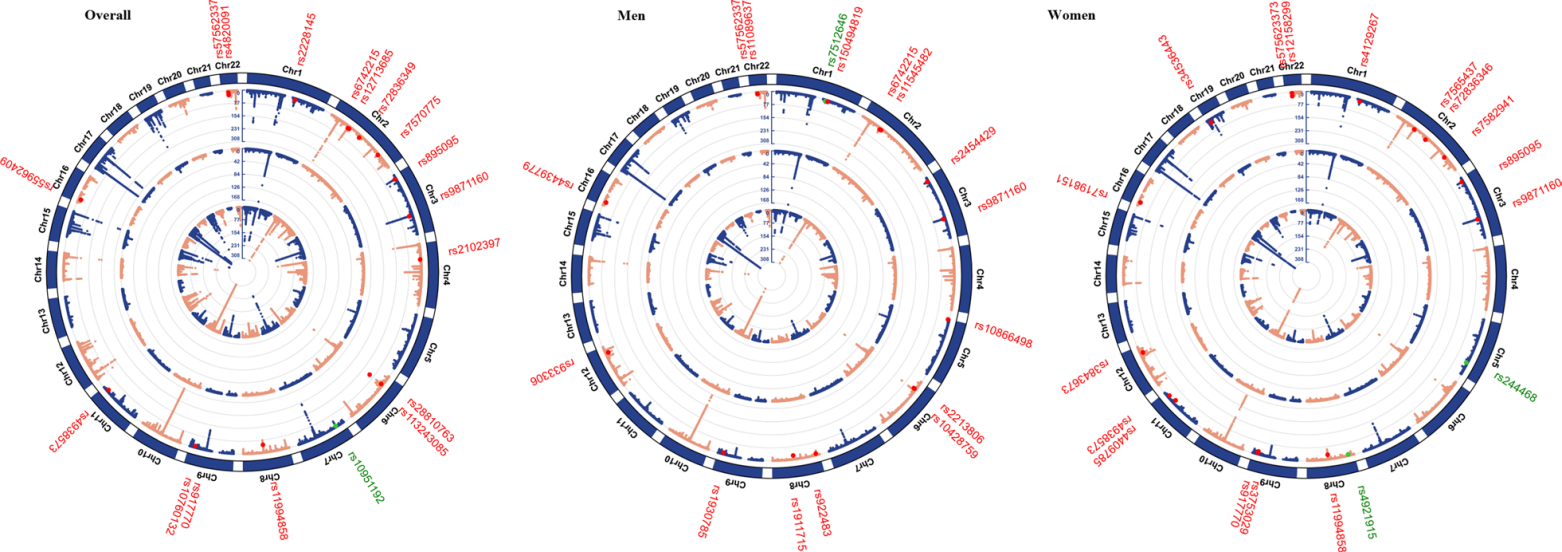
Cross-trait meta-analysis: BMI-adjusted sex hormone-binding globulin and rheumatoid arthritis (excluding MHC). Independent top-associated loci of sex hormone-binding globulin adjusted for BMI and rheumatoid arthritis were located in the inner and middle circles of the circular Manhattan plot, respectively. Results of cross-trait meta-analyses were listed in outer circles. Significant pleiotropic SNPs were presented by red spots, defined as variants with *P*_SHBGadjBMI_ < 1 × 10^–5^, *P*_RA_ < 1 × 10^–5^, and *P*_CPASSOC_ < 5 × 10^–8^. Novel pleiotropic SNPs detected for SHBG_adj_BMI with RA were marked by a green spot, and defined as a significant pleiotropic SNP neither reached genome-wide significance in a single trait (5 × 10^–8^ < *P*_SHBGadjBMI_ < 1 × 10^–5^, 5 × 10^–8^ < *P*_RA_ < 1 × 10^–5^, and *P*_CPASSOC_ < 5 × 10^–8^) nor in linkage-disequilibrium (LD) with previously identified SHBG- or RA-associated SNPs. MHC regions were excluded due to their complex LD pattern. Chr: chromosome; SHBG_adj_BMI: sex hormone-binding globulin adjusted for BMI. RA: rheumatoid arthritis

Motivated by the significant global and local genetic correlation, we further conducted a cross-trait meta-analysis to identify pleiotropic SNPs affecting both traits (Fig. 3 and Additional file 1 Table S7). Of the total 18 independent pleiotropic SNPs identified for SHBG_adj_BMI and RA, rs10951192 (*P*_CPASSOC_ = 8.99 × 10^–10^) was detected as a novel SNP and located in *JAZF1*, a gene implicated in transcriptional repression and inflammation suppression [31]. Additionally, we identified four “known” SNPs, seven “SHBG_adj_BMI-driven” SNPs, three “RA-driven” SNPs, and three “LD-tagged” SNPs. The sex-specific analyses revealed distinct pleiotropic SNPs underlying SHBG_adj_BMI and RA for men and women. For men, 16 independent pleiotropic SNPs were detected, among which rs7512646 (*P*_CPASSOC_ = 6.61 × 10^–10^) was novel, located in *IL6R*, a gene encoding a subunit of the interleukin 6 receptor complex [32] and participating in the immune response and autoimmune diseases [33]. For women, 18 independent pleiotropic SNPs were detected, two of which were novel: rs244468 (*P*_CPASSOC_ = 7.70 × 10^–10^), located in *ARHGAP26*, a gene encoding GTPase activating protein that regulates tumor immunity [34] and inflammation [35], as well as rs4921915 (*P*_CPASSOC_ = 3.59 × 10^–10^), an intergenic variant.

Replacing SHBG_adj_BMI with crude SHBG, a similar pattern of results was observed. In overall population, 70.0% of pleiotropic SNPs were either overlapped or in LD with SNPs identified using SHBG_adj_BMI. The proportion was 66.7% in men and 76.9% in women when analyzed by sex. Functional annotations of these pleiotropic SNPs are shown in (Additional file 1: Table S9, S10).

### Fine-mapping analysis

For each of the pleiotropic SNPs, we further determined a 99% credible set of causal SNPs, providing targets for future downstream experimental analysis (Additional file 1: Table S11). In general, we found 448 candidate causal SNPs across all shared loci between SHBG_adj_BMI and RA. Of these, three pleiotropic SNPs (rs113243085, rs917770, and rs575623373) showed a posterior probability of 1.00. In sex-specific analysis, 327 candidate causal SNPs were identified for men, and 385 for women.

### Transcriptome-wide association studies

After Bonferroni correction, single-trait TWAS identified 148 genes significantly associated with RA and 2,115 genes with SHBG_adj_BMI. Intersecting the single-trait TWAS results, we finally identified 16 pleiotropic genes (AC007389.1, AC007389.5, AC007613.1, AC012370.2, ACOXL-AS1, AP000553.3, CCDC116, FADS1, FADS2, PHF19, SNN, TMEM258, TRAF1, TRIM38, UBE2L3, and YDJC) shared by SHBG_adj_BMI and RA, and the corresponding figure was four for men and 11 for women (Additional file 1: Table S12). Notably, AC007389.1 and AC007389.5, the two pseudogenes, were originated from the same locus on chromosome 2. Moreover, seven pleiotropic genes identified through TWAS overlapped with nearby genes mapped by CPASSOC-identified SNPs.

### Bidirectional Mendelian randomization analysis

**Fig. 4:**
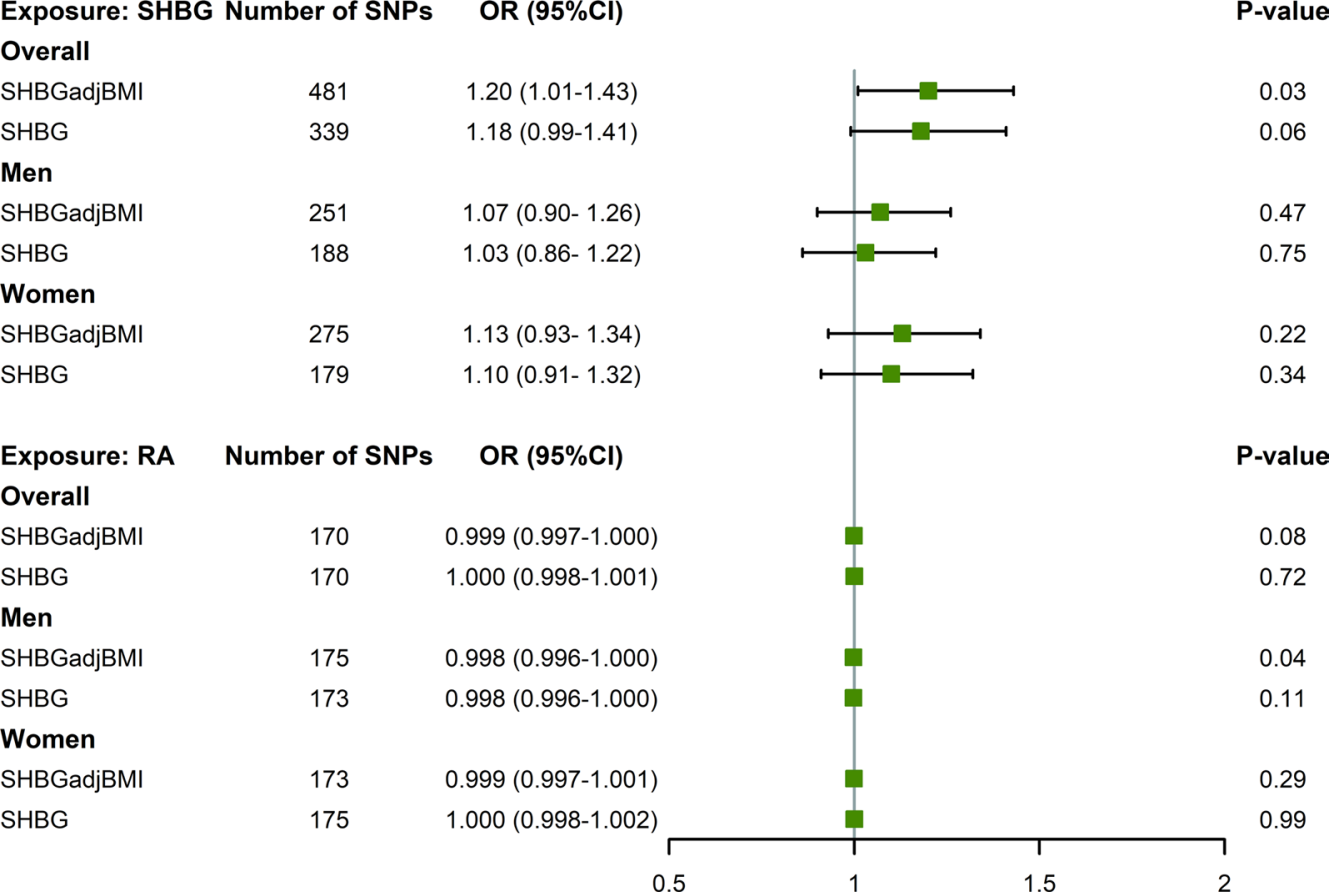
Causal associations: sex hormone-binding globulin and risk of rheumatoid arthritis in Mendelian randomization. Green squares represent odds ratio of outcomes per each SD increment in genetically predicted exposures. Bars represent 95% confidence intervals. The Mendelian randomization analysis was performed based on the inverse variance weighted approach. SHBG: sex hormone-binding globulin; SHBG_adj_BMI: sex hormone-binding globulin adjusted for BMI; RA: rheumatoid arthritis; SNP: Single nucleotide polymorphism; OR: odds ratio; CI: confidence interval

We performed a bidirectional two-sample MR analysis to evaluate a causal relationship. As shown in Fig. 4 and (Additional file 1: Table S13), a 20% increased risk of RA was observed per each SD (approximately 30.3 nmol/L) increment in genetically predicted SHBG_adj_BMI (IVW OR = 1.20, 95% CI = 1.01–1.43). The estimates remained directionally consistent in weighted median approach although with large uncertainty. In sex-specific analyses, the magnitude of effect size indicated potential disparities between men and women, with an OR of 1.13 (95% CI = 0.93–1.34) in women, and an OR of 1.07 (95% CI = 0.90–1.26) in men, although none of these estimates reached statistical significance.

We performed sensitivity analyses to verify the robustness of the results. The causal associations for SHBG_adj_BMI with RA were remained in overall population after correcting outliers (OR = 1.21, 95% CI = 1.04–1.38), removing confounding SNPs (OR = 1.49, 95% CI = 1.10–2.01) or palindromic SNPs (OR = 1.20, 95% CI = 0.99–1.45) (Additional file 1: Table S14). Consistent findings were also observed in MR-RAPS (Additional file 1: Table S15). Leave-one-out analysis indicated small fluctuations upon the exclusion of individual SNPs (Additional file 2: Figure S2).

On the contrary, no reverse causality was observed as genetically predicted RA did not seem to affect SHBG_adj_BMI either in overall or sex-specific analyses. The estimates were non-significant with effect sizes close to 1. These findings were further confirmed by Steiger directionality test (all *P* < 1 × 10^–99^) (Additional file 1: Table S13).

Replacing SHBG_adj_BMI with crude SHBG, similar but insignificant results were observed. Genetically predicted levels of crude SHBG may increase the risk of RA overall (IVW OR = 1.18, 95% CI = 0.99–1.41), but this trend was not observed either in men (IVW OR = 1.03, 95% CI = 0.86–1.22) or women (IVW OR = 1.10, 95% CI = 0.91–1.32).

Under the current sample size of outcome (97,173 with 23.00% of RA cases) and assuming an α of 0.05/3, given the phenotypic variance explained by SHBG_adj_BMI-associated IVs to be 0.178, our study had 80% power to detect a 6% change for the risk of RA with SHBG_adj_BMI. The corresponding effect sizes were 6% for men and 7% for women. For crude SHBG, with the phenotypic variance explained by index SNPs to be 0.132, our study had 80% power to detect a 7% change, with the corresponding effect sizes of 6% for men and 8% for women (Additional file 1: Table S16).

## Discussion

To the best of our knowledge, this is the first large-scale genome-wide cross-trait design that comprehensively investigates the shared genetic architecture underlying SHBG and RA. We found a significant shared genetic basis, both globally and locally, between SHBG_adj_BMI and RA overall. Such a genetic correlation could be further decomposed into horizontal pleiotropy and vertical pleiotropy, reflected by the multiple independent pleiotropic loci identified in cross-trait meta-analysis as well as the putative causal relationship confirmed by MR. Looking into men and women separately, most findings were replicated in both sexes, while sex-specific results were also observed, reflecting a distinct role of SHBG in affecting RA susceptibility across sex. Replacing SHBG_adj_BMI with crude SHBG, a largely similar yet less significant pattern of results was observed, corroborating main findings.

The SHBG-RA relationship remains largely inconclusive in clinical and epidemiological investigations [6–8]. A study suggested a lower SHBG level in RA patients [7], while other studies did not support this finding [6, 8]. Additionally, it is challenging to determine whether the decreased SHBG levels are caused by RA itself or by glucocorticoid treatment, a common method of treating RA which may result to decreased SHBG level [36]. Our findings, however, overcame the limitation of these studies and extended prior findings in several important ways. Through a comprehensive genome-wide cross-trait design, our study for the first time confirmed an intrinsic link underlying SHBG and RA, expanding beyond observational associations. The significant global and local SHBG-RA genetic correlations support a shared genetic basis unconfounded by environmental factors, of which magnitude ($r_{{\text{g}}}$ = 0.11) is noteworthy in comparison with figures estimated among autoimmune diseases, e.g., between RA and systemic lupus erythematosus ($r_{{\text{g}}}$ = 0.40, *P* = 6.01 × 10^–10^) or between RA and its well-established risk factor obesity ($r_{{\text{g}}}$ ranging from − 0.01 to 0.08, all *P* > 0.05) or between RA and its possible consequence bone mineral density ($r_{{\text{g}}}$ = − 0.059, *P* = 0.005) [37, 38]. Our study found a non-negligible genetic correlation of 11%, indicating a strong pathogenic link. The identification of multiple pleiotropic loci provides further evidence of a shared genetic basis. The validities of these pleiotropic SNPs were supported by fine-mapping analysis which identified three loci to be highly likely causal, and by TWAS analysis which overlapped with nearby genes mapped by CPASSOC-identified SNPs. We hereby provide a description of some of these pleiotropic genes. UBE2L3, a “known” pleiotropic gene, plays a role in both RA and sex hormone signaling [39, 40]. PHF19, TRAF1, and CCDC116 have been previously confirmed to be associated with RA [41–43]. However, their potential roles in SHBG were implicated by us. PHF19 is involved in the pathway of RNA polymerase I promoter opening and gene expression (transcription); TRAF1 is related to apoptosis and autophagy; CCDC116, a protein-coding gene located in centrosome, may be involved in RNA processing [44]. The level of SHBG may be regulated through these pathways. SNN, a protein-coding gene playing a role in the toxic effects of organotin [45] and endosomal maturation [46], has been proved to be linked to SHBG [11] and a number of other autoimmune diseases [47] including ankylosing spondylitis, psoriasis, ulcerative colitis, Crohn’s disease, and sclerosing cholangitis. AC007389.1 and AC007389.5, the two pseudogenes originating from the same locus, may interactively influence gene expression regulation. Finally, leveraging summary statistics of the hitherto largest GWAS(s) of both traits, the statistical power of our MR was greatly improved compared with previous MR [10]—involving a 37-fold increased number of IVs (481 vs. 13) and a six-fold augmented number of RA cases (22,350 vs. 4,017), we were able to detect a modest effect of 6–8% per standard deviation increment of genetically predicted SHBG with RA which previous studies were not well-powered to detect The negligible causal effect size observed in previous MR (OR = 1.003) is perhaps of limited clinical relevance in comparison with our findings (OR = 1.20). We were also able to determine the direction of association via bidirectional MR, suggesting SHBG may plays a pathogenic role in the development of RA rather than a secondary accompanying abnormality.

In addition to an overall landscape, distinct genetic architecture and biological mechanisms were identified for men and women separately through our sex-specific analysis. In addition to sex-specific shared genetic regions, nearly half of the pleiotropic loci we identified were sex-specific. We also observed potential differences in the pattern of pleiotropic genes between sexes, which may indicate the distinct pathways. Future studies are needed to validate the sex-specific role of these genes. TRIM38 and ZSCAN23 are men-specific genes. The former plays a role in modulating the severity of autoimmune disease [48] and negatively regulates inflammatory responses triggered by TLR3/4 and TNF/IL-1β [49] and nuclear factor (NF)-κB signaling [50]. ZSCAN23 has also been identified as an immune-related target gene [51]. In women, FADS2 is a member of the fatty acid desaturase gene family, which is involved in the pathways of alpha-linolenic acid metabolism and arachidonate biosynthesis III. Moreover, studies have shown that long-chain polyunsaturated fatty acids, synthesized by FADS2, have anti-inflammatory properties, and may be involved in regulating immune function [52, 53]. RGL2, a protein-coding gene, relates to the pathway of immune response antigen presentation by MHC class II. These identified genes may provide insights into the underlying sex-specific biological mechanisms.

Our analyses must be interpreted with caution. First, due to limited data availability, we could not examine RA subtypes characterized by anti-citrullinated antibody status or classify subgroups according to the severity of RA [41]. To our knowledge, nearly 90% of RA cases involved in our RA GWAS summary statistics were seropositive. Moreover, it appears that the seropositive and seronegative RA share heritability and have similar risk alleles outside of the MHC locus [54, 55]. Second, our study was conducted restricted to European ancestry populations. While this reduces population stratification and genetic heterogeneity, it also constrains the generalizability of findings to other ethnicities. Third, we need to be cautious in explaining the identified potential pleiotropic SNPs and genes through computational analysis. Further experimental validation is needed to strengthen the underlying biological mechanisms that we have detected. Fourth, adjusting for BMI in SHBG GWAS(s) could lead to BMI-associated SNPs being mistakenly identified as SHBG-associated SNPs (BMI as a collider), which may further violate the independence assumption of MR analysis (IVs are not associated with confounders). However, this collider bias has been minimized in the original GWAS study by discarding all loci which changed effect direction and/or had large changes in effect estimate and statistical significance when compared to the unadjusted model [11]. Moreover, we found a comparable effect of SHBG and SHBG_adj_BMI on RA (1.19 vs. 1.21) in MR analysis, which indicates a less likely biased result. Fifth, considering the uncertainty of the effect in a few sensitivity analyses and in sex-specific MR analysis, further validation may be needed to establish a robust causal relationship between SHBG_adj_BMI and RA.

## Conclusions

In conclusion, leveraging the hitherto largest genome-wide genetic data and advanced statistical approaches, the current study expands understanding of the observational association of SHBG with RA by providing evidence of genetic correlation, pleiotropic loci, and causal relationships. Our findings demonstrate an intrinsic, as well as a potential sex-specific link underlying SHBG and RA, and further shed novel light on biological mechanisms. Future studies are warranted to validate the function of the identified variants and genes, and to extend our findings on the diagnostic and therapeutic values of SHBG in RA.

### Supplementary Information

**Additional file 1: Table S1.** The characteristic of sex hormone-binding globulin adjusted for BMI associated index SNPs, their effect sizes with exposure and outcome, as well as their associations with potential confounders. **Table S2.** The characteristic of sex hormone-binding globulin adjusted for BMI associated index SNPs in men, their effect sizes with exposure and outcome, as well as their associations with potential confounders. **Table S3.** The characteristic of sex hormone-binding globulin adjusted for BMI associated index SNPs in women, their effect sizes with exposure and outcome, as well as their associations with potential confounders. **Table S4.** The characteristic of crude sex hormone-binding globulin associated index SNPs, their effect sizes with exposure and outcome, as well as their associations with potential confounders. **Table S5.** The characteristic of crude sex hormone binding globulin associated index SNPs in men, their effect sizes with exposure and outcome, as well as their associations with potential confounders. **Table S6.** The characteristic of crude sex hormone-binding globulin associated index SNPs in women, their effect sizes with exposure and outcome, as well as their associations with potential confounders. **Table S7.** Cross-trait meta-analysis between sex hormone-binding globulin adjusted for BMI and rheumatoid arthritis, excluding the MHC region (P_CPASSOC_ < 5×10^−8^, P_SHBGadjBMI_ < 1×10^−5^ and P_RA_ < 1×10^−5^). **Table S8.** Cross-trait meta-analysis between crude sex hormone-binding globulin and rheumatoid arthritis, excluding the MHC region (P_CPASSOC_ < 5×10^−8^, P_SHBG_ < 1×10^−5^ and P_RA_ < 1×10^−5^). **Table S9.** Functional annotation of SNPs shared between sex hormone-binding globulin adjusted for BMI and rheumatoid arthritis identified from the cross-trait meta-analysis, excluding the MHC region. **Table S10.** Functional annotation of SNPs shared between crude sex hormone-binding globulin and rheumatoid arthritis identified from the cross-trait meta-analysis, excluding the MHC region. **Table S11.** List of 99% credible set SNPs in each sex hormone-binding globulin adjusted for BMI and rheumatoid arthritis shared locus identified from fine-mapping analysis (*r*^2^ threshold = 0.6). **Table S12.** Shared significant genes between sex hormone binding globulin adjusted for BMI and rheumatoid arthritis from transcriptome-wide association studies using gene expressions across 49 GTEx tissues. **Table S13.** Bidirectional causal associations between genetically predicted sex hormone-binding globulin levels and risk of rheumatoid arthritis. **Table S14.** Sensitivity analysis of causal association between genetically predicted sex hormone-binding globulin levels and risk of rheumatoid arthritis. **Table S15.** Causal association between sex hormone-binding globulin levels and risk of rheumatoid arthritis based on the robust adjusted profile score (RAPS). **Table S16.** Power calculation in assumed and actual OR in our study.

**Additional file 2: Fig. S1.** Local genetic correlation between crude sex hormone-binding globulin and rheumatoid arthritis. Colored bars represent loci with significant local genetic correlation, covariance, and SNP-heritability after multiple testing adjustment. SHBG: sex hormone-binding globulin; RA: rheumatoid arthritis. **Fig. S2.** Leaving one SNP out at a time for the association between sex hormone-binding globulin and rheumatoid arthritis.

## Data Availability

All the data used in this study were from the publicly accessible GWAS summary statistics and can be accessed through the corresponding references presented in the main text.

## Abbreviations

BMI: Body mass index
CPASSOC: Cross-phenotype association analysis
GWAS: Genome-wide association studies
IVW: Inverse variance weighted
LD: Linkage disequilibrium
LDSC: LD-score regression
MAF: Minor allele frequency
MR: Mendelian randomization
MR-PRESSO: MR pleiotropy residual sum and outlier
MR-RAPS: Robust adjusted profile score
MVMR: Multivariable MR
PC: Principal component
PIP: Posterior inclusion probability
RA: Rheumatoid arthritis
SHBG: Sex hormone-binding globulin
SHBG_adj_BMI: BMI-adjusted SHBG
SNP: Single nucleotide polymorphism
TWAS: Transcriptome-wide association study
UKBB: UK Biobank
VEP: Variant Effect Predictor
ρ-HESS: Heritability estimation from summary statistics
