# Supplementary material for: A genome-wide cross-trait analysis identifies genomic correlation, pleiotropic loci, and causal relationship between sex hormone-binding globulin and rheumatoid arthritis
Source: Hum Genomics. 2023 Aug 29;17:81. doi: 10.1186/s40246-023-00528-x (PMC10466838; doi:10.1186/s40246-023-00528-x)
Supplement: Supplementary file 2 — Additional file 2: Fig. S1. Local genetic correlation between crude sex hormone-binding globulin and rheumatoid arthritis. Colored bars represent loci with significant local genetic correlation, covariance, and SNP-heritability after multiple testing adjustment. SHBG: sex hormone-binding globulin; RA: rheumatoid arthritis. Fig. S2. Leaving one SNP out at a time for the association between sex hormone-binding globulin and rheumatoid arthritis. [file 40246_2023_528_MOESM2_ESM.docx]

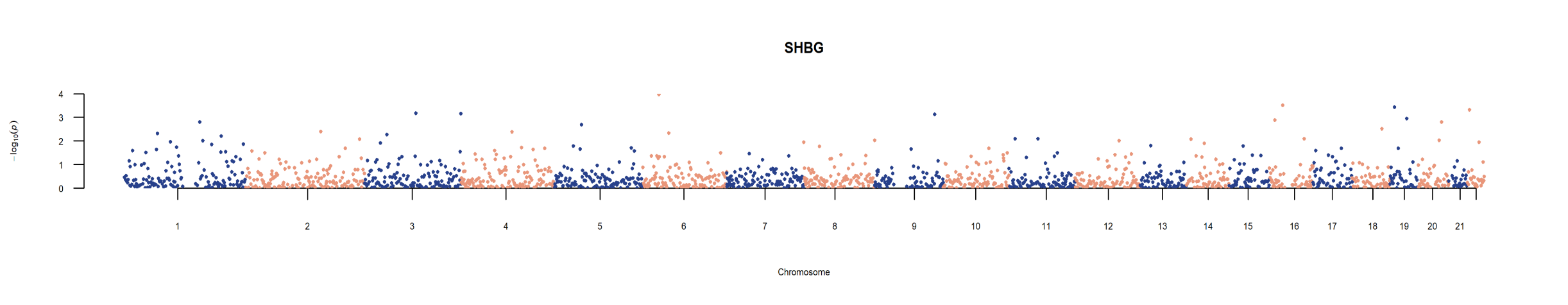

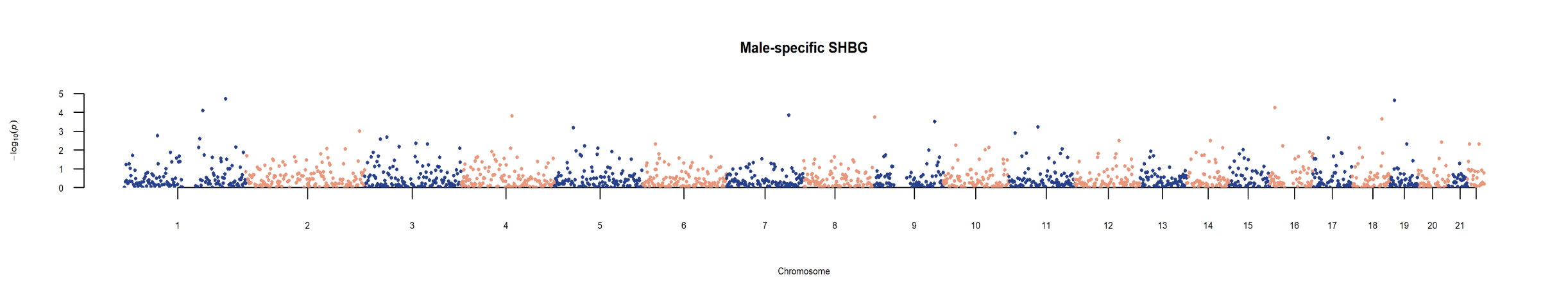

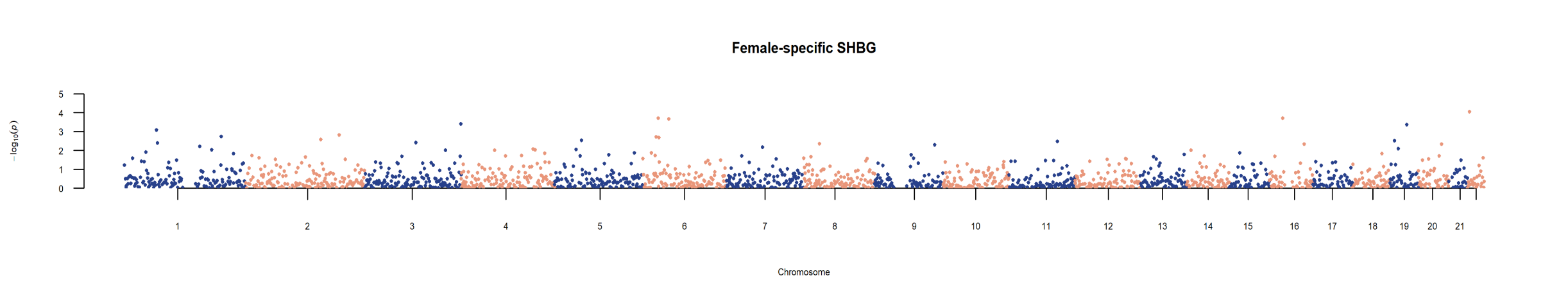


**SHBG and RA**

**Male-specific SHBG and RA**

**Female-specific SHBG and RA**

**Fig.S1 Local genetic correlation between crude sex hormone-binding globulin and rheumatoid arthritis.**

Colored bars represent loci with significant local genetic correlation, covariance, and SNP-heritability after multiple testing adjustment. SHBG: sex hormone-binding globulin; RA: rheumatoid arthritis


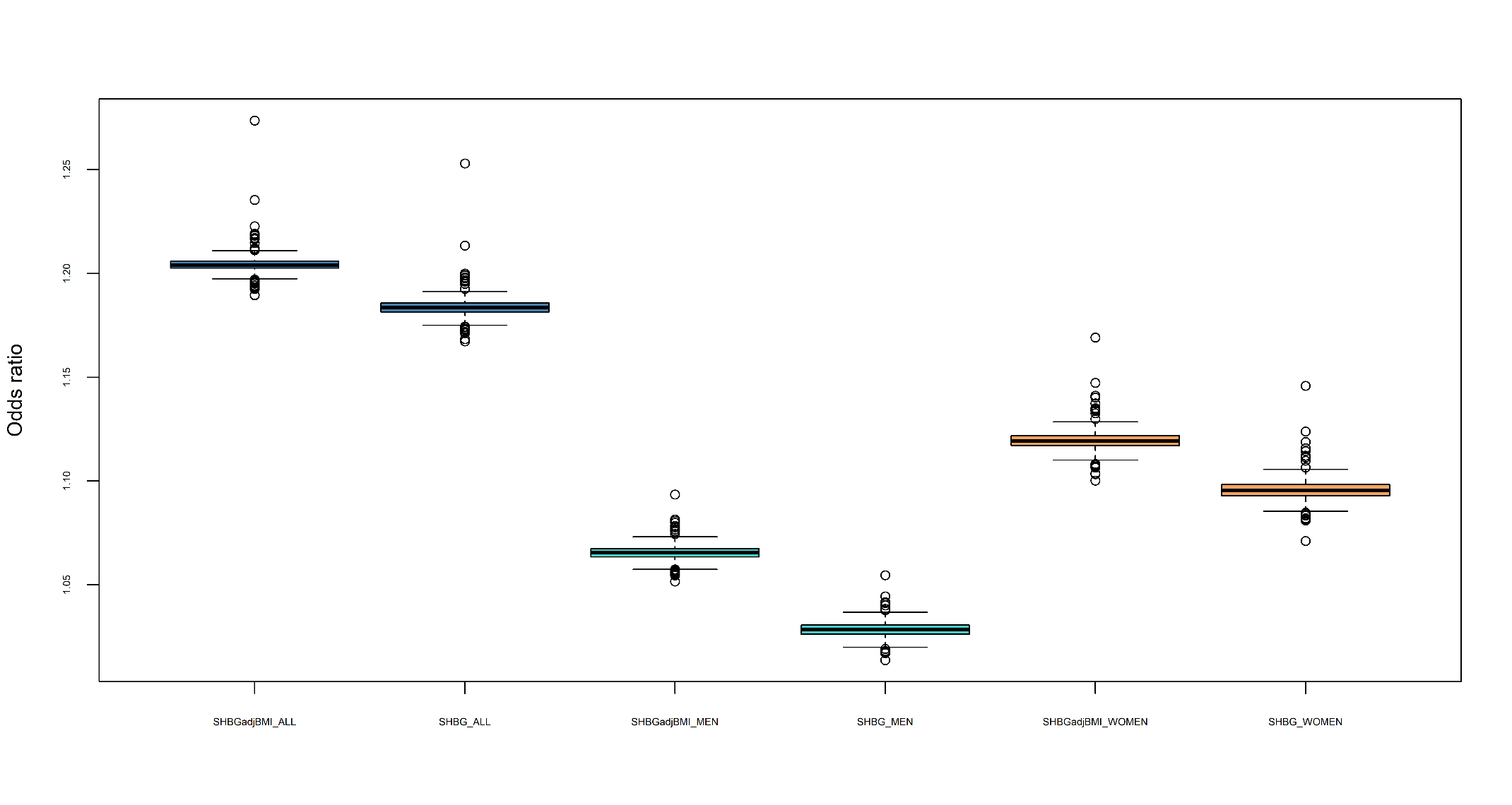


**Fig.S2 Leaving one SNP out at a time for the association between sex hormone-binding globulin and rheumatoid arthritis**
